# Supplementary material for: Effects of niche overlap on coexistence, fixation and invasion in a population of two interacting species
Source: R Soc Open Sci. 2020 Feb 19;7(2):192181. doi: 10.1098/rsos.192181 (PMC7062080; doi:10.1098/rsos.192181)
Supplement: Supplementary Information [file rsos192181supp1.pdf]

Supplementary Information: Effects of niche overlap on co-existence, fixation and invasion in a population of two interacting species

Minimal model of two interacting species and the derivation of the Lotka-Volterra model

As a minimal example, in this section we introduce a model of two interacting species whose growth is constrained by two secreted factors, inspired by the works of MacArthur and others [1–4]. Each species x_i has basal per capita birth rate β_i, death rate μ_i. Each species secretes soluble factors t_j at rates g_{ji}. Each factor t_i is degraded at a rate λ_i and affects the death rate of each bacterium linearly with the efficacy e_{ij}. Positive e_{ij} may correspond to metabolic wastes, toxins or anti-proliferative signals [5–10], while negative e_{ij} would describe growth factors or secondary metabolites [6, 11, 12]. The model kinetics is encapsulated in the following equations for the turnover of the species numbers:

$$\begin{aligned}\dot{x}_1 &= \beta_1 x_1 - \mu_1 x_1 - e_{11} t_1 x_1 - e_{12} t_2 x_1 \\ \dot{x}_2 &= \beta_2 x_2 - \mu_2 x_2 - e_{21} t_1 x_2 - e_{22} t_2 x_2, \end{aligned} \tag{1}$$

and the equations for the production and the degradation of the secreted factors:

$$\begin{aligned}\dot{t}_1 &= g_{11} x_1 + g_{12} x_2 - \lambda_1 t_1 \\ \dot{t}_2 &= g_{21} x_1 + g_{22} x_2 - \lambda_2 t_2. \end{aligned} \tag{2}$$

It is useful to recast Equations (1), (2) defining vectors $\vec{x} = (x_1, x_2)$ and $\vec{t} = (t_1, t_2)$, so that

$$\dot{\vec{x}} = \hat{R} \cdot \hat{X} \left(\vec{1} - \hat{E} \cdot \vec{t} \right) \quad \text{and} \quad \dot{\vec{t}} = \hat{L} \cdot \left(\hat{G} \cdot \vec{x} - \vec{t} \right), \tag{3}$$

where we have the matrices $\hat{X} = \begin{pmatrix} x_1 & 0 \\ 0 & x_2 \end{pmatrix}$, $\hat{L} = \begin{pmatrix} \lambda_1 & 0 \\ 0 & \lambda_2 \end{pmatrix}$, $\hat{R} = \begin{pmatrix} r_1 & 0 \\ 0 & r_2 \end{pmatrix} \equiv \begin{pmatrix} \beta_1 - \mu_1 & 0 \\ 0 & \beta_2 - \mu_2 \end{pmatrix}$, $\hat{G} = \begin{pmatrix} g_{11}/\lambda_1 & g_{12}/\lambda_1 \\ g_{21}/\lambda_2 & g_{22}/\lambda_2 \end{pmatrix}$, and $\hat{E} = \begin{pmatrix} e_{11}/r_1 & e_{12}/r_1 \\ e_{21}/r_2 & e_{22}/r_2 \end{pmatrix}$.

In many experimentally relevant systems, such as communities of microorganisms and cells, the timescale of production, diffusion, and degradation of secreted factors is on the order of minutes [13], whereas cell division and death occurs over hours [14, 15], and the dynamics of the turnover of the secreted factors can be assumed to adiabatically reach a steady state $\vec{t^*}$ given by $\vec{t^*} = \hat{G} \cdot \vec{x}$ [16–18]. In this approximation the dynamical equations for the species number reduce to

$$\dot{\vec{x}} = \hat{R} \cdot \hat{X} \left(\vec{1} - (\hat{E} \cdot \hat{G}) \cdot \vec{x} \right). \tag{4}$$

Written explicitly, this becomes the familiar generalized two-species competitive Lotka-Volterra system [2, 19–25]:

$$\begin{aligned}\dot{x}_1 &= r_1 x_1 \left(1 - \frac{x_1 + a_{12} x_2}{K_1} \right) \\ \dot{x}_2 &= r_2 x_2 \left(1 - \frac{a_{21} x_1 + x_2}{K_2} \right), \end{aligned} \tag{5}$$

where $\frac{1}{K_i} = \frac{e_{ii}g_{ii}}{r_i\lambda_i} + \frac{e_{ij}g_{ji}}{r_i\lambda_j}$ and $\frac{a_{ij}}{K_i} = \frac{e_{ii}g_{ij}}{r_i\lambda_i} + \frac{e_{ij}g_{jj}}{r_i\lambda_j}$; the matrix $(\hat{E} \cdot \hat{G})$ can be compactly written as $\begin{pmatrix} 1/K_1 & a_{12}/K_1 \\ a_{21}/K_2 & 1/K_2 \end{pmatrix}$.

The number of deterministically viable species is typically constrained by the number of limiting factors [3] (see the following section). Namely, if both matrices $\hat{E}$ and $\hat{G}$ are non-singular and invertible, Equations (3) possess a mixed fixed point given by $\vec{x}^* = (EG)^{-1}\vec{1}$. If this fixed point is stable and positive, it corresponds to the co-existence of the two species in two different (albeit potentially overlapping) ecological niches.

When the matrix $(\hat{E} \cdot \hat{G})$ is singular ($a_{12}a_{21} = 1$), the co-existence fixed point $\vec{x}^* = (EG)^{-1}\vec{1}$ does not exist, and the Equations (3) are satisfied only if the population of one (or both) of the species is zero. Biologically, this condition corresponds to the complete niche overlap between two species, whereby only one species can survive in the niche. (Of note, exclusion of one species by the other can also occur in non-singular cases, as discussed in the next section.) Nevertheless, even in the complete niche overlap case, multiple species can deterministically coexist within one niche if the matrix $(\hat{E} \cdot \hat{G})$ possesses a further degeneracy, $K_1/K_2 = a_{12} = 1/a_{21}$, corresponding to an additional symmetry in the interactions of the species with the constraining factors, as illustrated in the next paragraph.

These mathematical notions can be understood in a biologically illustrative example, when the matrix $\hat{E}$ is singular, so that $\det(\hat{E}) = 0$. Any singular 2×2 real matrix can be written in the general form $\hat{E} = \begin{pmatrix} \alpha & \alpha\beta \\ \alpha\gamma & \alpha\beta\gamma \end{pmatrix}$, where α, β and γ are arbitrary real numbers [26]. In this case Equation (1) becomes

$$\begin{aligned} \dot{x}_1 &= r_1 x_1 \big(1 - \alpha\,(t_1 + \beta t_2)\big) \\ \dot{x}_2 &= r_2 x_2 \big(1 - \gamma\alpha\,(t_1 + \beta t_2)\big), \end{aligned} \tag{6}$$

so that both secreted factors effectively act as one factor with concentration $t \equiv t_1 + \beta t_2$. With $\gamma \neq 1$ this corresponds to the classic notion of two species and only one limiting factor. The two equations cannot be simultaneously satisfied and the only solution of Equations (6) is either $x_1 = 0$ or $x_2 = 0$ (or both). This is one example of competitive exclusion due to competition within a single niche. Finally, when $\gamma = 1$ (corresponding to $a_{12} = 1/a_{21} = K_1/K_2$), both the species and the secreted factors are functionally identical, and the Equations (6) allow multiple solutions lying on the line in phase space defined by $\vec{x}^* = \hat{G}^{-1}\vec{t}^*$ and $1 = \alpha\,(t_1^* + \beta t_2^*)$ [21, 27]; in this case many different mixtures of the two species can be deterministically stable, depending on the initial conditions. These derivations above provide a mathematical definition and a biological illustration of the niche overlap between two interacting species, and can be extended to a general case of N species interacting via M factors [27].

Stability analysis of the LV model

Different regions of the parameter space of the Lotka-Volterra model, shown in Figure 1, have different biological interpretations [19, 28–31]. Parasitism, or predation/antagonism, occurs when $a_{12}a_{21} < 0$, with one species gaining from a loss of the other. In the strong parasitism regime where the positive a_{ij} is greater than one, the parasite/predator drives the prey to extinction deterministically, and the only stable point is the predator's fixed point (A or B). Conversely, weak parasitism allows co-existence of both species despite the detriment of one to the benefit of the other [19, 30].

Both $a_{ij} < 0$ corresponds to mutualistic/symbiotic interactions between the species [19, 28–30]. Weak mutualism is mathematically similar to weak competition in that it results in stable co-existence. Strong mutualism with $a_{12}a_{21} < -1$ results in population explosion. Detailed study of this regime lies outside of the scope of the present work (but see [32]).

The quadrant with both $a_{12} > 0$ and $a_{21} > 0$ corresponds to the competition regime. At strong competition with either a_{12} or a_{21} greater than one, either the system is bistable and possesses two single-species stable fixed points A and B with separate basins of attraction ($a_{12} > 1$ and $a_{21} > 1$) or one of the species deterministically outcompetes the other. The complete niche overlap regime is given by the line $a_{12}a_{21} = 1$. These regimes correspond to the classical competitive exclusion theory, together with the strong parasitism

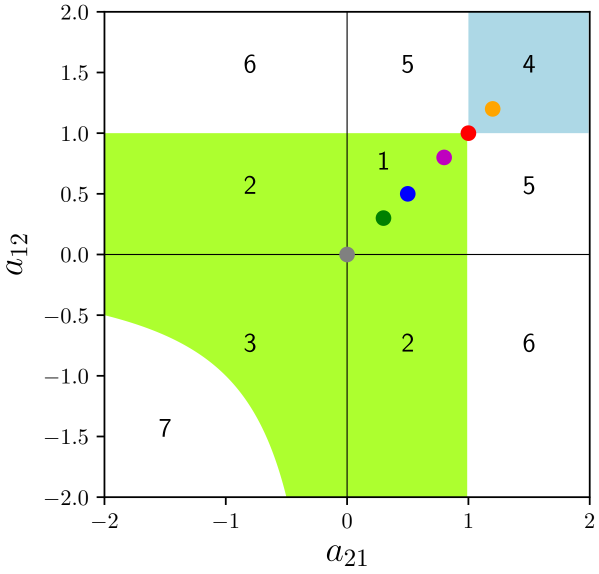

Figure 1: *Stability phase diagram of the co-existence fixed point for* $K_1 = K_2 = K$*.* The co-existence fixed point C of Equations (5) is stable in the green region and unstable in the blue region; in the white regions it is non-biological. Colored dots indicate the parameter range studied in the paper. The numbered regions correspond to different biological different regimes; see main text. Regions 4-6 correspond to competitive exclusion, with only single species fixed point A or B being stable (or both, in the bistable regime 5). In region 7 the populations experience unbounded growth. For the degenerate case $a_{12} = a_{21} = 1$, indicated by the red dot, the co-existence fixed point is replaced by a line of marginal stability, which we call the Moran line.

case. By contrast, weak competition where both $0 < a_{ij} < 1$ results in the stable co-existence at the mixed point C. In the special case $a_{12} = a_{21} = 1$ the stable fixed point degenerates into a neutral line of stable points, defined by $x_2 = K - x_1$, as shown in Figure 1.

Long-term deterministic stability of interacting species

Quite generally, the dynamics of a system of N asexually reproducing species that interact with each other only through M limiting factors (such as food, soluble signaling and growth/death factors, toxins, metabolic waste) and experience no immigration, can be described by the following system of differential equations for the species $x_1, ..., x_N$ and the limiting factor densities $f_1, ..., f_M$ [3, 27, 33]:

$$\dot{x}_i = \beta_i(\vec{f})x_i - \mu_i(\vec{f})x_i, \tag{7}$$

where $\vec{f}$ is the state of all factors that might affect the per capita birth rate $\beta_i(\vec{f})$ and the death rate $\mu_i(\vec{f})$ of the species i.

The density of a factor j in the environment, f_j, follows its own dynamical production-consumption equation

$$\dot{f}_j = g_j(\vec{f}, \vec{x}) - \lambda_j(\vec{f}, \vec{x})f_j \tag{8}$$

where g_j is a production-consumption rate that includes both the secretion and the consumption by the participating species as well any external sources of the factor f_j, and λ_j is its degradation rate. Alternatively,

for some abiotic constrains such as physical space or amount of sunlight, the concentration of the factor f_j can be set through a conservation equation of a form $f_j = c_j(\vec{f}, \vec{x})$ [3, 27].

The fixed points of the $N + M$ Equations (7) and (8) determine the steady state numbers of each of the N species and the corresponding concentrations of the M limiting factors. However, the structure of Equations (7) imposes additional constraints on the steady state solutions: at a fixed point $\beta_i(\vec{f}) = \mu_i(\vec{f})$ for each of the N species, which determines the steady state concentrations of the M limiting factors $\vec{f}$. However, if $N > M$, the system (7) of N equations is over-determined and typically does not have a consistent solution, unless the fixed point populations of $N - M$ of the species are equal to zero [3, 16, 27, 33, 34]. This reasoning provides a mathematical basis for the competitive exclusion principle, whereby the number of independent niches is determined by the number of limiting factors, and a system with M resources can sustain at most M species in steady state.

Nevertheless, as mentioned in the Introduction, the number of species at the steady state can exceed the number of limiting factors, when the N equations for the species are not independent and thus provide less than N constraints on the solutions. In this case, at steady state the populations of the non-independent species typically converge onto a marginally stable manifold on which each point is stable with respect to off-manifold perturbations but is neutral within the manifold [20, 27, 35–37].

Mean fixation time in the classical Moran model

Here we re-derive the mean fixation time for the Moran model [38], to provide context for the results of the main text. The Moran model results come about as one limit of the stochastic Lotka-Volterra system, when niche overlap is complete. In the classical Moran model, at each time step, an individual is chosen at random to reproduce from the total population of size K. In order to keep the population constant, another one is chosen at random to die. The probabilities that the number of individuals of species 1 increases ($b_M(n)$) or decreases ($b_M(n)$) by one in one time step are [38]:

$$b_M(n) = f(1-f) = (1-f)f = d_M(n) = \frac{n}{K}\left(1 - \frac{n}{K}\right) = \frac{1}{K^2} n(K-n), \tag{9}$$

where n is the number and f is the fraction of species 1 in the system (of total system size K).

The mean fixation time, $\tau(n)$, starting from some initial number n of species 1 is described by the following backward recursion equations [39]:

$$\tau(n) = \Delta t + d_M(n)\tau(n-1) + (1 - b_M(n) - d_M(n))\,\tau(n) + b_M(n)\tau(n+1),$$

where Δt is the time step. Substituting the values of the 'birth' and 'death' probabilities of species 1 from Equation (9) we get

$$\tau(n+1) - 2\tau(n) + \tau(n-1) = -\frac{\Delta t}{b_M(n)} = -\Delta t \frac{K^2}{n(K-n)}.$$

At $K \gg 1$, the Kramers-Moyal expansion in $1/K$ results in

$$\frac{\partial^2 \tau}{\partial n^2} = -\Delta t\, K \left(\frac{1}{n} + \frac{1}{K-n}\right).$$

Integrating, using the boundary conditions $\tau(0) = \tau(K) = 0$, gives

$$\tau(n) = -\Delta t\, K^2 \left(\frac{n}{K} \ln\left(\frac{n}{K}\right) + \frac{K-n}{K} \ln\left(\frac{K-n}{K}\right)\right). \tag{10}$$

For the initial condition analogous to the co-existence point, $n = K/2$, this gives

$$\tau = \Delta t\, K^2 \ln(2).$$

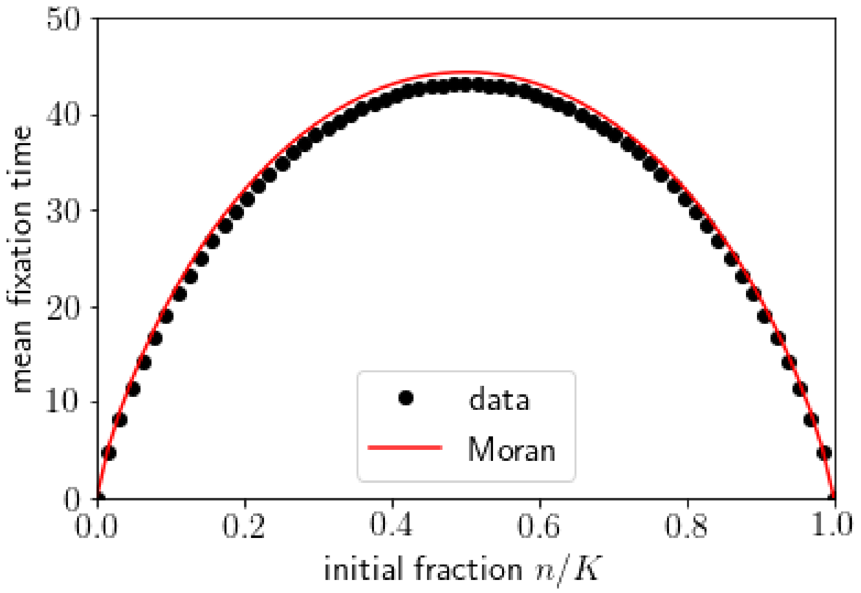

Figure 2: *The coupled logistic model agrees with the Moran model in the limit of complete niche overlap,* $a = 1$. The fixation time of the Moran model is shown in red; black are the numerical results of the the coupled logistic model for $a = 1$ using master equation approach. The population size of the Moran model is set equal to the carrying capacity of the corresponding coupled logistic model; $K = 64$.

For direct comparison with the coupled logistic model we need to consider that in the Moran model one time step Δt corresponds to one birth and one death event. Since the coupled logistic model spends most of its time near the WFM line $x_1 + x_2 = K$ we assume that on average

$$\Delta t \approx \frac{2}{b_1(x_1, K - x_1) + b_2(x_1, K - x_1) + d_1(x_1, K - x_1) + d_2(x_1, K - x_1)} \tag{11}$$

where b_i and d_i are the birth and death rates of the coupled logistic model. Since at the initial conditions the populations of each species are equal, and since $b_i(K/2, K/2) = d_i(K/2, K/2) = K/2$, we get $\Delta t \approx 1/K$. Therefore

$$\tau = \ln(2)\, K, \tag{12}$$

as in the main text. The fixation time of the Moran model agrees well with the results of the coupled logistic model for complete niche overlap, as shown in Figure 2.

Exact and approximate mean extinction time for a single species stochastic logistic model

In this section, we re-derive the known results for the extinction time of a single species logistic model.

Exact calculation. The mean extinction times for different initial states n_0 obey the usual backward recursion relation [39]

$$\tau[n_0] = \frac{1}{b(n_0) + d(n_0)} + \frac{b(n_0)}{b(n_0) + d(n_0)}\tau[n_0 + 1] + \frac{d(n_0)}{b(n_0) + d(n_0)}\tau[n_0 - 1]. \tag{13}$$

where $b(n) = r\,n$ and rate $d(n) = r\,n\frac{n}{K}$ are the birth and death rates of the process, respectively. The

equation above can be rewritten as

$$\tau[n_0+1]-\tau[n_0]=\left(\tau[1]-\sum_{i=1}^{n_0} q_i\right) S_{n_0}, \tag{14}$$

where

$$q_0=\frac{1}{b(0)} \quad q_1=\frac{1}{d(1)}, \tag{15}$$

$$q_i=\frac{b(i-1)\cdots b(1)}{d(i)d(i-1)\cdots d(1)}=\frac{1}{d(i)}\prod_{j=1}^{i-1}\frac{b(j)}{d(j)},\ i>1,$$

and

$$S_i=\frac{d(i)\cdots d(1)}{b(i)\cdots b(1)}=\prod_{j=1}^{i}\frac{d(j)}{b(j)}. \tag{16}$$

The logistic process becomes extinct in finite time, and the extinction time can be thus written as follows [39]:

$$\tau[n_0]=\sum_{i=1}^{\infty} q_i+\sum_{j=1}^{n_0-1} S_j \sum_{i=j+1}^{\infty} q_i. \tag{17}$$

Evaluating this sum with $b(n)=rn$, $d(n)=rn^2/K$ [40] and the initial condition $n_0=K\gg 1$ gives the asymptotic limit

$$r\,\tau\simeq\frac{1}{K}e^K \tag{18}$$

to leading order [41]. This result agrees with the more general approach of [42].

Fixation time of the coupled logistic model in the independent limit

Here we calculate the mean fixation time in the independent limit of the coupled logistic model. The fixation occurs when either of the species goes extinct. Denoting the probability distribution of the extinction times for either of the independent species as $p(t)$ and its cumulative as $f(t)=\int_{s=0}^{t} p(s)ds$, the probability that *either* of the species goes extinct in the time interval $[t,t+dt]$, is

$$p_{min}(t)dt=\Big(p(t)\left(1-f(t)\right)+\left(1-f(t)\right)p(t)\Big)dt=2p(t)\left(1-f(t)\right)dt. \tag{19}$$

The mean time to fixation $\langle t\rangle$ is

$$\langle t\rangle=\int_0^\infty dt\,t\,p_{min}(t). \tag{20}$$

As shown in Figure 3, the probability distribution of the fixation times of a single species is dominated by the exponential tail. It can be approximated as

$$p(t)=\alpha e^{-\alpha t}, \quad f(t)=1-e^{-\alpha t} \tag{21}$$

with $\frac{1}{\alpha}\simeq\frac{1}{K}e^K$ from the previous section. Finally, we obtain for the mean time to fixation of the two-species logistic model

$$\langle t\rangle=\int_0^\infty dt\,t\,2\alpha e^{-2\alpha t}=\frac{1}{2\alpha}. \tag{22}$$

which leads to the equation $\tau\simeq\frac{1}{2K}e^K$ in the main text.

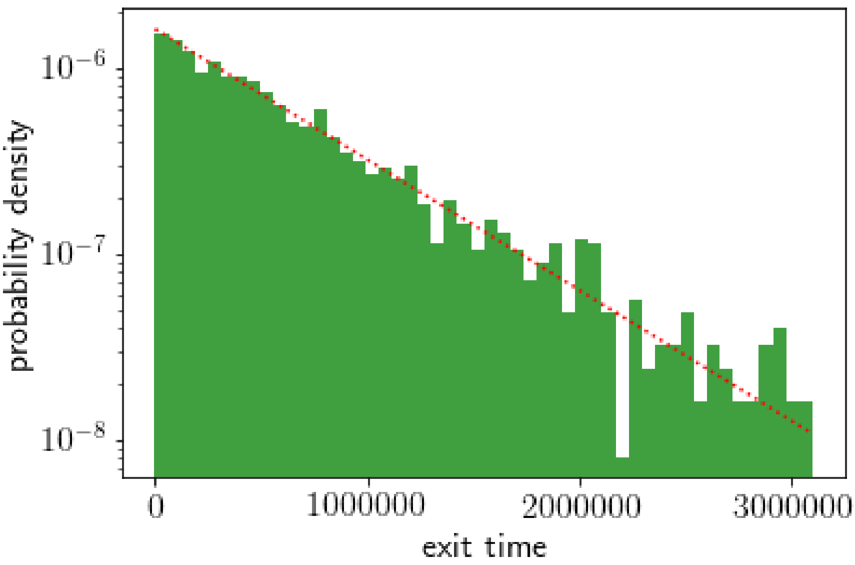

Figure 3: *Extinction time distribution of the logistic model is dominated by a single exponential tail.* The bulk of the probability density is well described by an exponential distribution with the same mean, shown in the red dotted line. Data are generated using using the Gillespie algorithm for $K = 16$. For higher carrying capacities the assumption of exponentially distributed times becomes even more accurate.

Fokker-Planck approximation and the pseudo-potential

In this section we derive an analytical approximation for the results in Figure 2 in the main text, using Fokker-Planck approximation. The Fokker-Planck approximation to the coupled logistic system studied herein takes its traditional form [43]:

$$\begin{aligned}\frac{dP}{dt} &= -\partial_1[(b_1 - d_1)P] - \partial_2[(b_2 - d_2)P] + \frac{1}{2}\partial_1^2[(b_1 + d_1)P] + \frac{1}{2}\partial_2^2[(b_2 + d_2)P] \\ &= -\sum_i \partial_i F_i P + \frac{1}{2}\sum_{i,j} \partial_i \partial_j D_{ij} P \end{aligned} \tag{23}$$

where F is the drift (or force) vector and D is the diffusion matrix (in this case diagonal). Here, under symmetric conditions and non-dimensionalization by r, $F_i = \frac{n_i}{K}(K - n_i - a_{ij}n_j)$ and $D_{ii} = \frac{n_i}{K}(K + n_i + a_{ij}n_j)$.

In general, Equation (23) cannot be reduced to diffusion in a potential $U(\vec{n})$ with an equilibrium distribution function $P(\vec{n}) \sim \exp(U(\vec{n}))$. The condition of zero flux at equilibrium, $J_i = F_i P - 1/2 \sum_j \partial_j D_{ij} P = 0$, would require [39, 43]

$$\partial_i \log P = \sum_k (D^{-1})_{ik} \big(2F_k - \sum_j \partial_j D_{kj}\big) \equiv -\partial_i U,$$

However, for consistency it also requires $\partial_j\left(-\partial_i U\right) = \partial_i\left(-\partial_j U\right)$ [39, 43]. It is easy to show that this is not upheld for the two directions unless $a_{12} = a_{21} = 0$ and the system can be decomposed into two one-dimensional logistic systems.

Instead, we define the pseudo-potential as:

$$U(n_1, n_2) \equiv -\ln\left[P_{ss}(n_1, n_2)\right]. \tag{24}$$

where $P_{ss}(n_1, n_2)$ is a quasi-stationary probability distribution function [44]. We calculate $P_{ss}(n_1, n_2)$ in the approximation to the Fokker-Planck Equation (23) linearized about the deterministic co-existence fixed point

[39, 43, 45]. The linearized equation is [39, 43]

$$\partial_t P = -\sum_{i,j} A_{ij}\partial_i(n_j - n_j^*)P + \frac{1}{2}\sum_{i,j} B_{ij}\partial_i\partial_j(n_i - n_i^*)(n_j - n_j^*)P \tag{25}$$

where $A_{ij} = \partial_j F_i|_{\vec{n}=\vec{n}^*}$ and $B_{ij} = D_{ij}|_{\vec{n}=\vec{n}^*}$. The quasi-equilibrium solution to Equation (25) is $P_{ss} = \frac{1}{2\pi}\frac{1}{|C|^{1/2}}\exp[-(\vec{n}-\vec{n}^*)^T C^{-1}(\vec{n}-\vec{n}^*)/2]$, a Gaussian centered on the co-existence point and with a variance given by the covariance matrix $C = B \cdot A^{-1}/2$ in the symmetric case $a_{12} = a_{21} = a$, $K_1 = K_2 = K$ [43]. In this case the diagonal term of C is $\frac{1}{1-a^2}K$ and gives the variance of a species about its mean value. The off-diagonal, which corresponds to the covariance between the two species, is $-\frac{a}{1-a^2}K$. Thus the Pearson correlation coefficient between the two species is $-a$.

For the initial condition at the co-existence fixed point and assuming that the system escapes towards fixation once it reaches one of the axial fixed points $(0, K)$ or $(K, 0)$, from Equation (24) the well depth is proportional to carrying capacity K, being

$$\Delta U = \frac{(1-a)}{2(1+a)}K. \tag{26}$$

In a Kramers' type approximation, the escape time from the pseudo-potential well scales as $\sim \exp(\Delta U)$ [46], reproducing the exponential scaling of the extinction time with K, observed numerically. Moreover, the Fokker-Planck approximation also shows that the exponential scaling disappears as niche overlap a approaches unity, in accord with the numerical results in the main text. The correlation between the two species goes to negative one in this parameter limit, such that they are entirely anti-correlated. Whereas the well has a single lowest point at the co-existence fixed point for partial niche overlap, at $a = 1$ the potential shows a trough of equal depth going between the two axial fixed points. This is the Moran line, along which diffusion is unbiased; diffusion away from the Moran line is restored, as the system is drawn toward the bottom of the trough. Because everywhere along the Moran line is equally likely, the probability cannot be normalized, and the linearization approximation fails. This is to be expected, as it is an expansion about a fixed point, but the fixed point is replaced by the Moran line in the Moran limit of $a = 1$.

Breaking the parameter symmetries

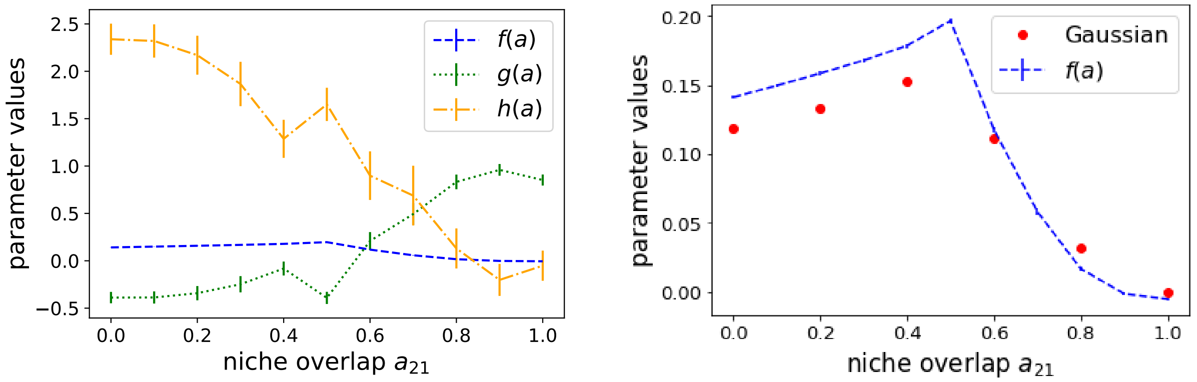

Figure 4: *Breaking the symmetry in a. Left:* Dashed lines denote the functions in the $\tau = e^{h(a_{21})}K^{g(a_{21})}e^{f(a_{21})K}$ ansatz obtained from the fit to the numerical data. come from fitting the ansatz to generated data. *Right:* The right panel compares the results of the ansatz fit with Kramers'/Fokker-Planck estimate of the fixation time.

The main text treats the symmetric case of $K_1 = K_2 \equiv K$ and $a_{12} = a_{21} \equiv a$. Here, we extend our results to the asymmetric case, where the symmetry between the parameters is broken. Although the exponential

scaling of the fixation time with the system size (except at the Moran line), persists also in the asymmetric case although the exponential dependence can be much weaker in some of the asymmetric cases.

Figure 4 shows the dependence of the fixation time on the niche overlap a_{21} while keeping $a_{12} = 0.5$ for $K_1 = K_2 \equiv K$, using the similar ansatz we apply the same $\tau = e^{h(a_{21})}K^{g(a_{21})}e^{f(a_{21})K}$. As the niche overlap a_{21} changes from 0 to 1, the location of the co-existence fixed point shifts from $(K/2, K/4)$ to $(K, 0)$. Accordingly, the fixation time starting from the fixed point maintains its exponential scaling with carrying capacity up until $a_{21} = 1$, where the fixed time is equal to zero, as reflected in the shape of the of $h(a_{21})$. Notably, in the asymmetric case the exponential scaling function $f(a_{21})$ is much weaker compared to the symmetric case, partially because the fixed point is located closer to an axis that in the symmetric case even at $a_{21} = 0$. The right panel of Figure 4 shows the comparison of the results of the ansatz fit with the estimates of the exponential part of the fixation time using Kramers'/Fokker-Planck pseudo-potential described in the previous section that explains the observed trends of $f(a_{21})$.

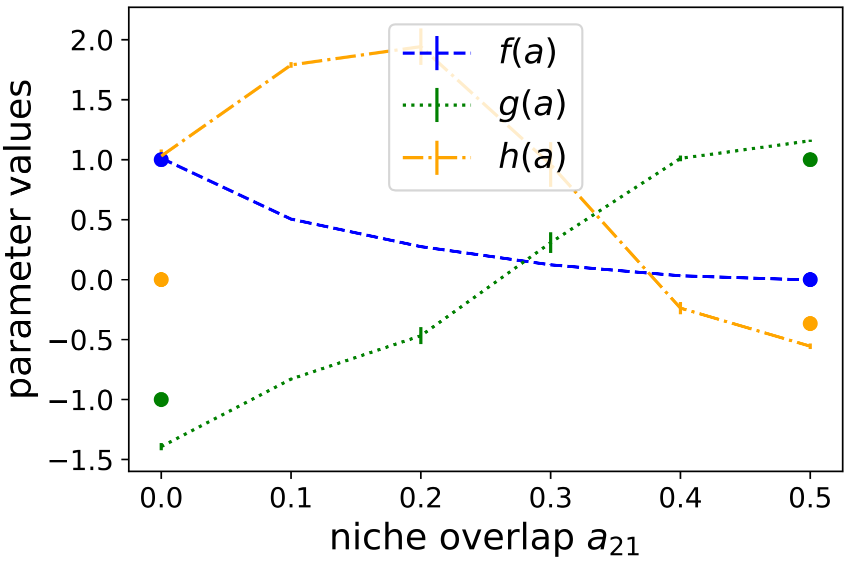

Figure 5: *Breaking the symmetry in K.* As in Figure 2 in the main text, lines come from fitting the ansatz to generated data. The exponential dependence is non-zero except at the appearance of the Moran line at $a_{21} = 1/2$. The extreme points are the expected asymptotic values.

Next let us consider breaking the symmetry such that the Moran line can still be recovered. The carrying capacity symmetry is broken, such that $K_2 = 2K_1$. The two species are still independent when $a_{12} = a_{21} = 0$, but in this case the Moran line exists when $a_{12} = 2$ and $a_{21} = 1/2$. Figure 5 shows the results when the symmetry is broken both in the carrying capacity and the niche overlap. It shows the change in the fixation time as a function of the niche overlap a_{21} for $K_2 = 2K_1 \equiv K$ while the niche overlaps change along the line where $a_{12} = 4a_{21}$, starting from the independent case $a_{12} = a_{21} = 0$ to $a_{12} = 2$ and $a_{21} = 1/2$ where the system reaches its corresponding Moran line. The observed behaviour is very similar to that shown in the symmetric case, with the exponential dependence transitioning smoothly to zero at the Moran line.

I uphold the conclusion that only at the Moran line will fixation be fast; when the system parameters are even slightly off those niche overlap values which balance the carrying capacities and allow for the Moran line to exist, the fixation is exponential in the carrying capacity, to the point that the two species effectively co-exist.

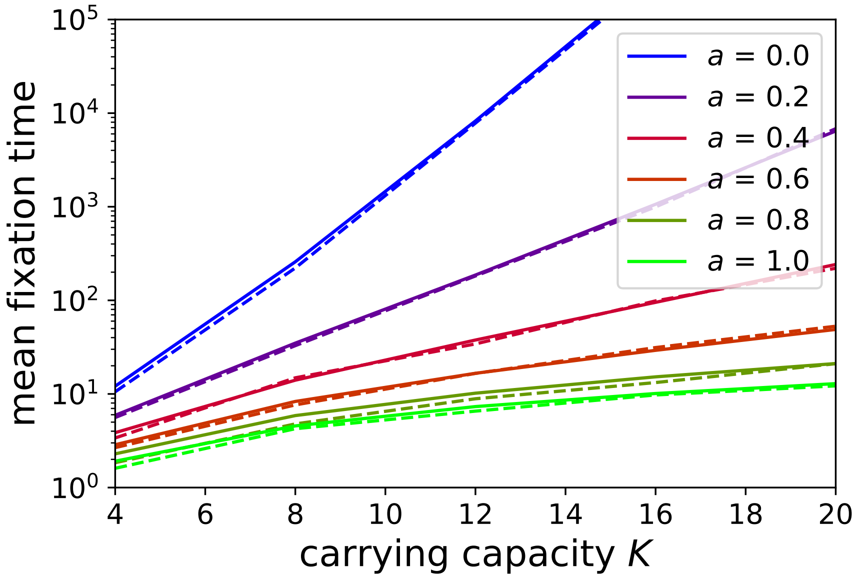

Figure 6: *Directly solving the (truncated) master equation agrees with Gillespie simulations.* Solid lines come from directly solving the backwards master equation by inverting the transition matrix, after a cutoff has been applied to the matrix to make it finite. Dashed lines are each an average of a hundred realizations of the stochastic process, as simulated using the Gillespie algorithm.

Comparison with the Gillespie algorithm

In this section, we verify the numerical results for the mean fixation times obtained using the master equation approach with the truncated transition matrix, agrees with the exact sampling of the fixation process using Gillespie algorithm [47], as shown in Figure 6. fixating. To ensure accuracy of the mean times to 0.1% or better we have chosen the cutoff value $C_K = 5K$ although this is largely excessive and even $C_K = 2K$ is sufficient for accurate calculation of the fixation times all but the smallest carrying capacities.

Map of a, K showing Co-existence versus Fixation

In the main text we state that since biological system sizes are typically large, a fixation time that scales exponentially with carrying capacity effectively implies co-existence. However, some systems have only a few competing members, as in nascent cancers or plasmids in a single cell. We want to get a better sense of when the exponential scaling is relevant, especially since for those systems with almost complete niche overlap the exponential scaling is slow. To this end we compare the expected mean fixation time with that of the Moran model. The ansatz $e^{h(a)}K^{g(a)}e^{f(a)K}$ is fit to the data and then used to estimate the fixation time at a variety of parameter values. This time is compared to the fixation time of a Moran model with the same carrying capacity. In figure 7 the shaded region represents those parameter combinations for which the estimated fixation is faster than the corresponding Moran model. As is evident, a carrying capacity of forty is fully sufficient to allow for effective co-existence of two species which are not identical in their niches. Even for systems with a smaller carrying capacity, unless the two species are similar they are expected to co-exist for long times before fixation. For example, for a niche overlap of $a = 0.9$ the threshold carrying capacity is around $K = 13$, which is not relevant for most ecological systems, but could be relevant for instance in plasmid dynamics [48] or mitochondrial disease in yeast [49]. The odd curvature at $K = 5$ comes from an extrapolation of the ansatz to low numbers; for a system with such a small carrying capacity, the simplifying assumptions underlying the model are expected to break down.

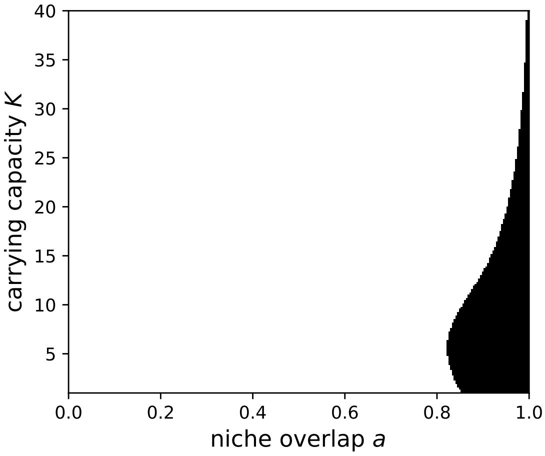

Figure 7: *Parameter space in which fixation is fast.* The white area shows where two species are expected to effectively co-exist, while the black shading identifies the regime where fixation is faster than a similar Moran model. Fixation is estimated by extrapolating the ansatz parameter fits to the a, K parameter space.

Invasion into a one-dimensional deterministic logistic model

In this section we estimate the time a deterministic single logistic system would take to grow from one organism close to its carrying capacity.

$$\begin{aligned}
\tau_s &= \int dt = \int_{x_o}^{x_f} dx \frac{1}{\dot{x}} \\
&= \frac{1}{r} \int_{x_o}^{x_f} dx \frac{K}{x(K-x)} = \frac{1}{r} \int_{x_o}^{x_f} dx \left(\frac{1}{x} - \frac{1}{K-x} \right) \\
&= \frac{1}{r} \ln \left[\frac{x}{K-x} \right] \Big|_{x_o}^{x_f} \\
&= \frac{1}{r} \ln \left[\frac{x_f(K-x_o)}{x_o(K-x_f)} \right].
\end{aligned}$$

If we assume $x_0 = 1$, $K \gg 1$, and $x_f = (1-\epsilon)K$ then this becomes

$$\tau_s \approx \frac{1}{r} \ln \left[\frac{(1-\epsilon)K}{\epsilon} \right].$$

If we further assume that $\epsilon \ll 1$ we can conclude

$$\tau_s \approx \frac{1}{r} \left(\ln[K] - \ln[\epsilon] \right) \tag{27}$$

and so expect the invasion time to grow logarithmically with carrying capacity. These results are well-known in the literature [41, 50].

References

[1] Caperon J. Population growth in micro-organisms limited by food supply. Ecology. 1967;48(5):715–722.

[2] MacArthur R. Species Packing and Competitive Equilibrium for Many Species. Theoretical population biology. 1970;11.

[3] Armstrong RA, McGehee R. Competitive Exclusion. Am Nat. 1980;115(2):151–170.

[4] Chesson P. MacArthur ' s Resource Model. Theoretical Population Biology. 1990;37:26–38.

[5] Jacob C. Cytokines and anti-cytokines. Current opinion in immunology. 1989;2(2):249–257.

[6] Maplestone RA, Stone MJ, Williams DH. The evolutionary role of secondary metabolitesa review. Gene. 1992;115(1):151–157.

[7] Van Melderen L, De Bast MS. Bacterial toxin-Antitoxin systems: More than selfish entities? PLoS Genet. 2009;5(3).

[8] Rankin DJ, Turner La, Heinemann Ja, Brown SP. The coevolution of toxin and antitoxin genes drives the dynamics of bacterial addiction complexes and intragenomic conflict. Proceedings Biological sciences / The Royal Society. 2012 sep;279(1743):3706–15.

[9] Shen P, Fillatreau S. Antibody-independent functions of B cells: a focus on cytokines. Nature Reviews Immunology. 2015;15(7):441.

[10] Wynn TA. Type 2 cytokines: mechanisms and therapeutic strategies. Nature Reviews Immunology. 2015;15(5):271.

[11] Reya T, Morrison SJ, Clarke MF, Weissman IL. Stem cells, cancer, and cancer stem cells. Nature. 2001;414(6859):105.

[12] Wink M. Evolution of secondary metabolites from an ecological and molecular phylogenetic perspective. Phytochemistry. 2003;64(1):3–19.

[13] Belle A, Tanay A, Bitincka L, Shamir R, OShea EK. Quantification of protein half-lives in the budding yeast proteome. Proceedings of the National Academy of Sciences. 2006;103(35):13004–13009.

[14] Powell EO. Growth rate and generation time of bacteria, with special reference to continuous culture. Journal of general microbiology. 1956;15(3):492–511.

[15] Lenski RE, Rose MR, Simpson SC, Tadler SC. Long-Term Experimental Evolution in Escherichia coli. I. Adaptation and Divergence During 2,000 Generations. The American Naturalist. 1991;138(6):1315.

[16] Posfai A, Taillefumier T, Wingreen NS. Metabolic Trade-Offs Promote Diversity in a Model Ecosystem. Physical Review Letters. 2017;118(2):028103.

[17] Assaf M, Meerson B. WKB theory of large deviations in stochastic populations. Journal of Physics A: Mathematical and Theoretical. 2017;50(26):263001.

[18] Chotibut T, Nelson DR. Population Genetics with Fluctuating Population Sizes. J Stat Phys. 2017;167(3-4):777–791.

[19] Chotibut T, Nelson DR. Evolutionary dynamics with fluctuating population sizes and strong mutualism. Physical Review E - Statistical, Nonlinear, and Soft Matter Physics. 2015;92(2):022718.

[20] Dobrinevski A, Frey E. Extinction in neutrally stable stochastic Lotka-Volterra models. Physical Review E - Statistical, Nonlinear, and Soft Matter Physics. 2012;85(5):1–14.

[21] Constable GWA, McKane AJ. Models of genetic drift as limiting forms of the Lotka-Volterra competition model. Phys Rev Lett. 2015;114(3):1–5.

[22] Bomze IM. Lotka-Volterra Equation and Replicator Dynamics: A Two-Dimensional Classification. Biological Cybernetics. 1983;211:201–211.

[23] Levin SA. Community equilibria and stability, and an extension of the competitive exclusion principle. The American Naturalist. 1970;104(939):413–423.

[24] Czuppon P, Traulsen A. Fixation probabilities in populations under demographic fluctuations. Journal of mathematical biology. 2018;77(4):1233–1277.

[25] Young G, Belmonte A. Explicit probability of fixation formula for mutual competitors in a stochastic population model under competitive trade-offs. arXiv preprint arXiv:180906917. 2018;.

[26] Larson R. Elementary linear algebra. Nelson Education; 2016.

[27] McGehee R, Armstrong RA. Some mathematical problems concerning the ecological principle of competitive exclusion. Journal of Differential Equations. 1977;23(1):30–52.

[28] Neuhauser C, Pacala SW. An Explicitly Spatial Version of the Lotka-Volterra Model with Interspecific Competition. Ann Appl Probab. 1999;9(4):1226–1259.

[29] Cox JT, Merle M, Perkins E, et al. Coexistence in a two-dimensional Lotka-Volterra model. Electronic Journal of Probability. 2010;15:1190–1266.

[30] May RM. Stability and complexity in model ecosystems. vol. 6. Princeton university press; 2001.

[31] Abrams PA. Density-independent mortality and interspecific competition: a test of Pianka's niche overlap hypothesis. The American Naturalist. 1977;111(979):539–552.

[32] Meerson B, Sasorov PV. Noise-driven unlimited population growth. Physical Review E. 2008;78(6):060103.

[33] Armstrong RA, McGehee R. Coexistence of species competing for shared resources. Theoretical Population Biology. 1976;9:317–328.

[34] Fisher CK, Mora T, Walczak AM. Habitat Fluctuations Drive Species Covariation in the Human Microbiota. arXiv preprint arXiv:151000198. 2015;p. 1–18.

[35] Case TJ, Casten R. Global stability and multiple domains of attraction in ecological systems. The American Naturalist. 1979;113(5):705–714.

[36] Lin YT, Kim H, Doering CR. Features of Fast Living: On the Weak Selection for Longevity in Degenerate Birth-Death Processes. Journal of Statistical Physics. 2012;148(4):646–662.

[37] Antal T, Scheuring I. Fixation of strategies for an evolutionary game in finite populations. Bulletin of Mathematical Biology. 2006;68(8):1923–1944.

[38] Moran P. The Statistical Processes of Evolutionary Theory. Oxford: Clarendon Press; 1962.

[39] Nisbet RM, Gurney WSC. Modelling Fluctuating Populations. Toronto: John Wiley & Sons; 1982.

[40] Gradshteyn IS, Ryzhik IM. Table of integrals, series, and products. Academic press; 1965.

[41] Lande R. Risks of Population Extinction from Demographic and Environmental Stochasticity and Random Catastrophes. The American Naturalist. 1993;142(6):911–927.

[42] Lambert A. The branching process with logistic growth. The Annals of Applied Probability. 2005;15(2):1506–1535.

[43] Van Kampen NG. Stochastic Processes in Physics and Chemistry. North-Holland, Amsterdam: North Holland; 1992.

[44] Zhou JX, Aliyu MDS, Aurell E, Huang S. Quasi-potential landscape in complex multi-stable systems. J R Soc Interface. 2012;9(77):3539–3553.

[45] Grasman J. The expected extinction time of a population within a system of interacting biological populations. Bulletin of mathematical biology. 1996;58(3):555–568.

[46] Hänggi P, Talkner P, Borovec M. Reaction rate theory - 50 years after Kramers. Rev Mod Phys. 1990;62(2):251–341.

[47] Gillespie DT. Exact Stochastic Simulation of couple chemical reactions. The Journal of Physical Chemistry. 1977;81(25):2340–2361.

[48] del Solar G, Giraldo R, Ruiz-Echevarria MJ, Espinosa M, Diaz-Orejas R. Replication and control of circular bacterial plasmids. Microbiol Mol Biol Rev. 1998;62(2):434–464.

[49] Taanman JW. The mitochondrial genome: structure, transcription, translation and replication. Biochimica et Biophysica Acta (BBA)-Bioenergetics. 1999;1410(2):103–123.

[50] Parsons TL. Invasion probabilities, hitting times, and some fluctuation theory for the stochastic logistic process. Journal of mathematical biology. 2018;77(4):1193–1231.
